# OCT4 expression mediates partial cardiomyocyte reprogramming of mesenchymal stromal cells

**DOI:** 10.1371/journal.pone.0189131

**Published:** 2017-12-07

**Authors:** Gustavo Yannarelli, Natalia Pacienza, Sonia Montanari, Diego Santa-Cruz, Sowmya Viswanathan, Armand Keating

**Affiliations:** 1 Cell Therapy Program, Princess Margaret Cancer Centre, University Health Network, Toronto, ON, Canada; 2 Laboratorio de Regulación Génica y Células Madre, Instituto de Medicina Traslacional, Trasplante y Bioingeniería (IMeTTyB), Universidad Favaloro/CONICET, Buenos Aires, Argentina; Georgia Regents University, UNITED STATES

## Abstract

Mesenchymal stem/stromal cells (MSCs) are in numerous cell therapy clinical trials, including for injured myocardium. Acquisition of cardiomyocyte characteristics by MSCs may improve cardiac regeneration but the mechanisms regulating this process are unclear. Here, we investigated whether the pluripotency transcription factor OCT4 is involved in the activation of cardiac lineage genetic programs in MSCs. We employed our established co-culture model of MSCs with rat embryonic cardiomyocytes showing co-expression of cardiac markers on MSCs independent of cell fusion. Bone marrow-derived MSCs were isolated from transgenic mice expressing GFP under the control of the cardiac-specific α-myosin heavy chain promoter. After 5 days of co-culture, MSCs expressed cardiac specific genes, including Nkx2.5, atrial natriuretic factor and α-cardiac actin. The frequency of GFP+ cells was 7.6±1.9%, however, these cells retained the stromal cell phenotype, indicating, as expected, only partial differentiation. Global OCT4 expression increased 2.6±0.7-fold in co-cultured MSCs and of interest, 87±5% vs 79±4% of MSCs expressed OCT4 by flow cytometry in controls and after co-culture, respectively. Consistent with the latter observation, the GFP+ cells did not express nuclear OCT4 and showed a significant increase in OCT4 promoter methylation compared with undifferentiated MSCs (92% vs 45%), inferring that OCT4 is regulated by an epigenetic mechanism. We further showed that siRNA silencing of OCT4 in MSCs resulted in a reduced frequency of GFP+ cells in co-culture to less than 1%. Our data infer that OCT4 expression may have a direct effect on partial cardiomyocyte reprogramming of MSCs and suggest a new mechanism(s) associated with MSC multipotency and a requirement for crosstalk with the cardiac microenvironment.

## Introduction

Multipotent mesenchymal stromal cells (MSCs) can be easily obtained from different tissue sources including bone marrow (BM), adipose tissue and umbilical cord [1–3]. Because of their anti-inflammatory, immunomodulatory, angiogenic and anti-apoptotic effects, MSCs have been used to treat various diseases [4–6]. Pre-clinical models of cardiac injury show that MCSs can facilitate myocardial repair and angiogenesis [7–9]. Clinical trials data, however, indicate that transplantation of BM-derived cells results in a modest 2–3% absolute increase in left ventricular ejection fraction [10–12]. A better understanding of possible mechanisms mediating cardiac regeneration will enable the use of different strategies to enhance the potency of MSCs and improve clinical outcomes. Paracrine action of MSCs, rather than direct regeneration by differentiation into cardiomyocytes, more likely explains the hemodynamic improvement as engraftment in the host myocardium is not required [13–16]. Consistent with this notion, we found that BM-derived MSCs acquire cardiac specific markers but retain MSCs properties when co-cultured with rat embryonic cardiomyocytes (RECs) [17]. In addition, we showed that most donor cells express cardiac markers but retain the stromal phenotype when lodged in the hearts of mice subjected to experimental acute myocardial infarction (AMI) [18]. Although MSCs demonstrate varying degrees of cell lineage plasticity, cardiomyocyte reprogramming of MSCs is partial both *in vitro* and *in vivo*, further supporting a paracrine mechanism of action. The partial reprogramming that results from the interaction of MSCs with the cardiac microenvironment, however, may be an important additional mechanism to engender myocardial repair. In this regard, some reports have shown that cardiovascular lineage commitment of MSCs enhances their therapeutic effects [19–21]. We also found that MSCs derived from the perivascular tissue of the umbilical cord (HUCPVCs) exhibit a greater degree of cardiomyocyte reprogramming than do BM-MSCs and provide improved cardiac function in an AMI model after intra-myocardial injection [22] but not when administered systemically [15]. These data infer a link between partial cardiomyocyte reprogramming and cardiac regenerative potential, as HUCPVCs showed improved benefit compared with BM-MSCs only when donor cells located mainly in the infarct area. Thus, it is important to better understand the mechanisms mediating this partial reprogramming that underlie the interaction between MSCs and the cardiac microenvironment. Interestingly, we recently found that HUCPVCs present higher levels of stromal progenitors and exhibit enhanced OCT4 expression compared with BM-MSCs [23]. The biological characteristics of the neonatal origin of HUCPVC tissue may position OCT4 as a key factor in mediating MSCs multipotency.

OCT4 (Pou5f1) is a well-known transcription factor that regulates the self-renewal and pluripotency of embryonic stem cells (ESCs) [24,25]. In addition, OCT4 is essential for nuclear reprogramming as it can induce the reprogramming of fibroblasts into ESC-like induced pluripotent stem cells (iPSCs), either alone or in combination with other factors [26,27]. Thus, OCT4 is considered the master pluripotency factor that controls other nuclear regulators of the pluripotency network, including NANOG and SOX2 [28]. Greco et al. also found that MSCs express OCT4 and suggested a similar regulatory role for this factor, although its level of expression was significantly lower than in ESCs [29]. Consistent with this notion, we recently showed that OCT4 promoter methylation is significantly higher in MSCs vs ESCs, suggesting a possible epigenetic mechanism for the limited plasticity of MSCs [23]. Whether OCT4 expression mediates MSC multipotency, however, has not been clearly demonstrated.

In the present study, we investigated the role of the pluripotency factor OCT4 in the partial cardiomyocyte reprogramming of MSCs using our established co-culture model with RECs. We found that OCT4 expression is directly related to the ability of MSCs to acquire a partial cardiomyocyte phenotype. Moreover, MSCs must first gain OCT4 (de-differentiate) before being able to commence cardiomyocyte reprogramming, a mechanism that resembles the reprogramming process of adult stem cells. This study provides a novel mechanism that further supports our previous data inferring a link between partial cardiomyocyte differentiation and the regeneration potential of MSCs [15,22,23].

## Materials and methods

### Bone marrow-derived MSCs

Animal procedures conformed to the US National Institute of Health’s guidelines for the care and use of laboratory animals with approval from the Animal Care and Use Committee of the Ontario Cancer Institute and Universidad Favaloro/CONICET. B-a-Fvb mice were generated by using a transgenic vector containing a green florescent protein (GFP) coding sequence flanked by the full-length mouse α-myosin heavy chain (α-MHC) promoter [17]. Bone marrow MSCs were collected from the femur and tibia of individual adult B-a-Fvb mice. Mononuclear cells were separated by Ficoll density gradient (Ficoll-Paque PLUS, GE Healthcare-Amersham Biosciences). Cells were suspended in MSC medium, consisting of Dulbecco’s modified Eagle’s medium-low glucose (DMEM-LG) (Life technologies) supplemented with 10% fetal bovine serum (FBS) and 1% antibiotic-antimitotic solution (Life technologies). Cells were plated at 20x10^6^ cells/75 cm^2^ and incubated at 37°C in a humidified incubator with 5% CO_2_. When the adherent layer reached near confluence (70–80%), the cells were detached using 0.25% trypsin and 1 mM EDTA, and serially passaged at 4000 cells/cm^2^ every 5–7 days until fourth passage (P4) cells were obtained. Surface phenotype analysis and lineage differentiation into adipocytes, osteocytes and chondrocytes were done to confirm that cells displayed the properties consistent with the ISCT minimal requirements for the definition of MSCs [30].

### Co-culture of BM-MSCs with rat embryonic cardiomyocytes (RECs)

Cardiomyocytes were isolated from rat embryos (day 20) and cultured on the basis of the procedures previously described [17]. Briefly, ventricles were minced and digested using trypsin (0.08% w/v) and collagenase type 2 (0.035% w/v; both Worthington Biochemical Corporation). Pre-plating for 60 min was performed to eliminate contaminating fibroblasts. Nonadherent cells were collected and seeded on gelatin-coated plates (Sigma-Aldrich) in DMEM:F12 (1:1; Gibco) supplemented with 5% FBS and 10% horse serum (Gibco). After 48 h, fourth passage BM-MSCs were plated onto the primary RECs cultures and co-cultured for up to 5 days. This time frame for co-culture was chosen based on previous results [17]. To determine cell fusion events, only male RECs were used in the co-culture experiments with BM-MSCs isolated from female mice. MSCs were also co-cultured with isolated embryonic rat lung, kidney, and liver cells.

### Quantitative RT-PCR

Total RNA was extracted from cells using Trizol reagent (Invitrogen) as described by the manufacturer. One microgram was reverse transcribed into cDNA using random primers and MultiScribe RT (High-Capacity cDNA Reverse Transcription Kit, Applied Biosystems). Mouse specific primers were designed to detect the expression of pluripotency factors and cardiac specific markers in the co-culture system (S1 Table). RT-PCR reactions were performed to evaluate the primers design and specific amplification products were confirmed by automatic DNA sequencing (see supplementary material for methodology details). For quantitative RT-PCR, samples were assayed in triplicate using Power SYBR Green master mix on a 7900HT real-time PCR system (Applied Biosystems) with the following conditions: 1 cycle for 10 min at 95°C, 40 cycles with 95°C for 15 s and 60°C for 60 s, followed by a melting curve analysis. Results were analyzed using the Relative Quantification (ΔΔCt) method [31]. The threshold cycle (Ct) values were normalized against the reference gene glyceraldehyde-3-phosphate dehydrogenase (GAPDH). The data were calculated using the formula 2-ΔΔCt and are presented as the fold change in gene expression normalized and relative to the untreated control.

### Western blotting

Total cellular protein extracts were prepared from untreated MSCs and CD44^+^-sorted MSCs after the co-culture as described by Maniatis et al. [32]. Protein concentrations were determined using the micromethod of Bradford (Bio-Rad, CA). Samples (60 μg total protein) were separated on a 10% SDS-PAGE gel and transferred to a HyBlot CL film (Denville Scientific, NJ, USA). Membranes were blocked using 1X PBST with 5% (w/v) non-fat dried milk for 1 h and hybridized overnight at 4°C with primary antibodies. Membranes were incubated with anti-rabbit or anti-rat HRP-conjugated secondary antibodies and were washed five times for 5 min with TBST buffer. Bound antibodies were visualized using ECL Plus Western Blotting detection system (GE Healthcare) according to the manufacturer’s instructions. Primary antibodies used were anti-OCT4 (1:800, Abcam, ab19857), anti-SOX2 (1:1500, Abcam, ab97959), anti-NANOG (1:800, Abcam, ab80892), and anti-β-Tubulin (1:1000, Abcam, ab6161).

### Flow cytometry analysis and cell sorting

BM-derived MSCs from B-a-Fvb mice were co-cultured with RECs as described above. After 5 days, cells were trypsinized, washed in PBS and resuspended in FACS buffer (PBS + 2% FBS). Cells were stained with an anti-mouse CD44 antibody linked APC (1:50, eBioscience, 17–0441) for 30 min, which did not cross react with rat CD44 and was therefore used to distinguish mouse from rat cells in the co-culture system. For OCT4 expression analysis, cells were then washed and fixed/permeabilized using 4% paraformaldehyde and methanol. After permeabilization, cells were stained for OCT4 using anti-OCT4 (1:50, Abcam, ab19857) and Alexa Fluor 594-conjugated secondary antibody (1:100, Molecular Probes, A-21207). Flow cytometry was performed on a FACS Calibur flow cytometer (Becton Dickinson). For cell sorting, cells were washed after CD44 staining and cell sorting was performed under sterile conditions using BD FACS Aria (University Health Network Flow Cytometry Facility). GFP detection was carried out with a 530/30 nM band pass filter and a 505 nM long pass filter. CD44-APC was detected with a 660/20 nM band pass filter and no long pass filter. Sorting was done at a pressure of 20 psi with a 100 μm nozzle and the cells were divided into three populations: (1) GFP+ and CD44+, (2) GFP- and CD44+ and (3), CD44-. The sorted GFP+/CD44+ and GFP-/CD44+ cells were centrifuged (1000 rpm, 10 min) and analyzed using immunostaining and bisulfite genomic sequencing as described below.

### Immunostaining

Immunostaining techniques were used to determine the frequency of cardiomyocyte differentiation (GFP+/Col IV+ cells) in the co-culture experiments. After 5 days of co-culture, cells were fixed in 4% paraformaldehyde/PBS for 10 min, permeabilized with 0.2% Triton X-100/PBS for 10 min, and then blocked with 5% FBS/PBS for 1 h. Cells were then incubated overnight at 4°C with a goat polyclonal anti-GFP-linked FITC antibody (1:100, Abcam) and a rabbit anti-mouse collagen type IV (1:100, BioDesign).

The secondary antibody for collagen type IV was an Alexa Fluor 555 conjugated anti-rabbit IgG (1:200, Molecular Probes). For immunostaining of sorted cells, cells were spun down on gelatin-coated slides using Cytospin 4 (Thermo Fisher Scientific) and then fixed with 4% paraformaldehyde. Cardiac troponin T was stained by using a monoclonal antibody to cardiac troponin T (BioDesign). Collagen type IV was stained with a rabbit anti-mouse collagen type IV (BioDesign). In both cases, the secondary antibody used was linked with Alexa Fluor 555 (Molecular Probes). For OCT4 staining, cells were incubated overnight at 4°C with primary antibodies against OCT4 (1:250, Abcam, ab19857). After incubation, cells were washed and incubated for 1 h at room temperature with the secondary antibody Alexa Fluor 594-conjugated donkey anti-rabbit IgG (1:250, Molecular Probes, A-21207). Nuclei were stained with DAPI (DAPI ProLong Gold, Invitrogen). Detection of fluorescent signals was performed using confocal laser scanning microscopy (Fluoview FV1000, Olympus).

### OCT4 silencing

The vector used for the stable siRNA transfection was the pSilencer 4.1 CMV that employs a CMV promoter and a puromicyn resistance gene as a mechanism to select for transfected cells (Life Technologies). To silence OCT4 expression, we used the following oligonucleotides 5′-GATCCGAGCACGAGTGGAAAGCAATTCAAGAGATTGCTTTCCACTCGTGCTCCTA-3′ and 5′-AGCTTAGGAGCACGAGTGGAAAGCAATCTCTTGAATTGCTTTCCACTCGTGCTCG-3′ that were annealed and cloned into the BamHI and HindIII sites of the pSilencer-4.1-CMV puro vector (siOCT4). The construction was confirmed by DNA sequencing. As the negative control, a plasmid encoding a scrambled hairpin siRNA (siScr) whose sequence is not found in the mouse database was used (Life Technologies). MSCs were transfected with the different siRNA constructs using Metafectene Easy+ (Biontex) according to the manufacturer's instructions. 24 h post transfection, stable transfected cells were selected using puromycin (2.5 μg/ml).

### Bisulfite genomic sequencing

Genomic DNA was purified using DNeasy Tissue Kit (Quiagen) from a pool of three independent samples of GFP-/CD44+ and GFP+/CD44+ FACS-sorted cells after co-culture. Total DNA was treated with sodium bisulfite using the MethylCode Bisulfite Conversion Kit (Invitrogen). Samples were amplified by nested-PCR with primers designed to specifically recognize the OCT4 promoter region only in bisulfite-converted DNA [33] (S1 Table). PCR products were gel-purified with QIAquick Gel Extraction Kit (Quiagen) and then cloned into bacteria by pGEM-T easy cloning (Invitrogen). Sequences of 10–15 bacterial clones per sample examined were analyzed using BISMA software [34]. Bisulphite conversion efficiency of non-CpG cytosines was higher than 95% for all individual clones for each sample.

### Statistical analysis

All data are presented as mean±SD. Statistical significance was assessed by Student’s *t* test and one-way ANOVA with Tukey’s multiple comparison post-test. *p* values < 0.05 were defined as statistically significant.

## Results

### MSCs express cardiomyocyte specific markers after co-culture with RECs

**Fig 1:**
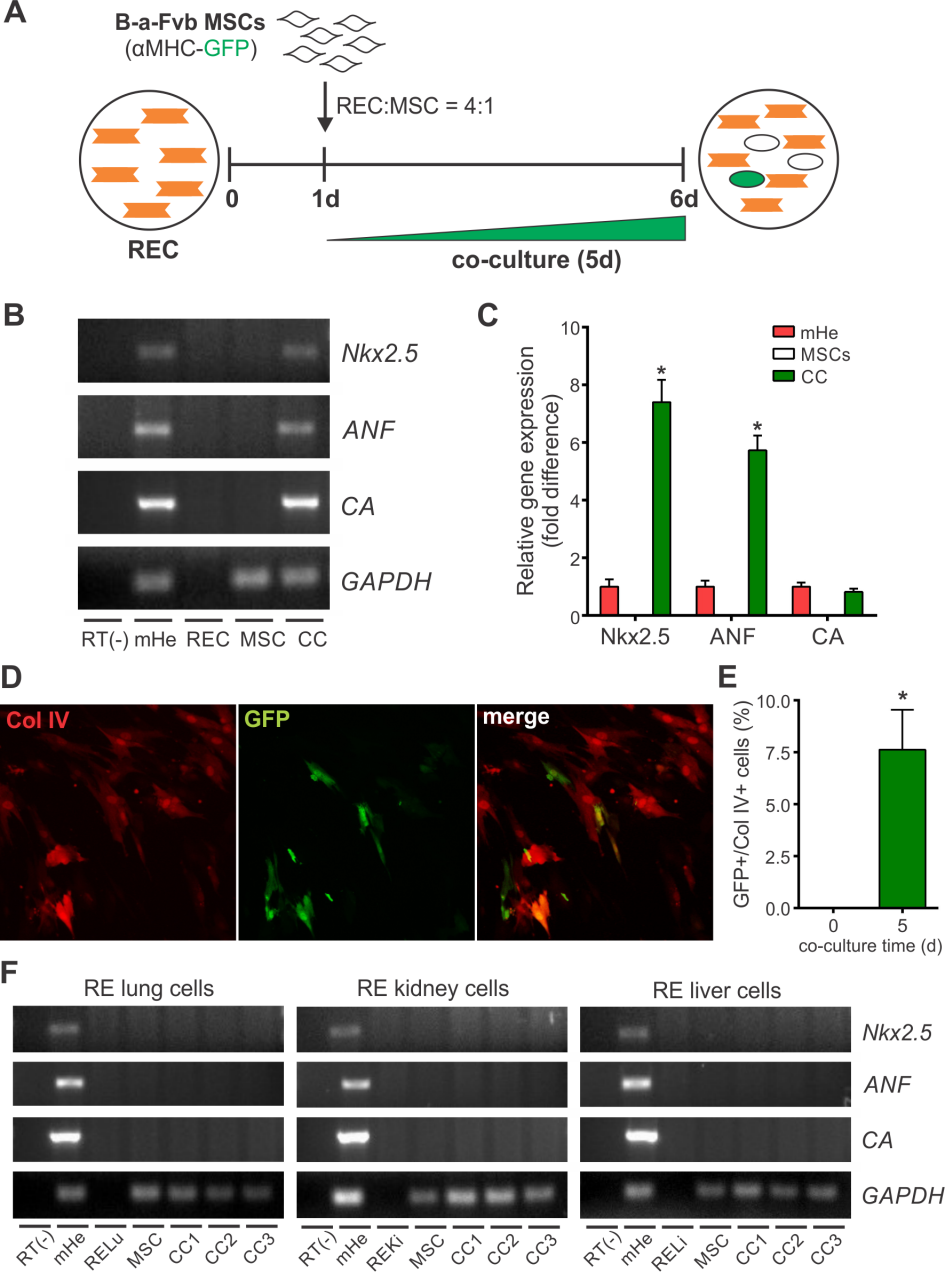
BM-MSCs acquire specific cardiomyocyte markers after co-culture with rat embryonic cardiomyocytes (RECs) but not when co-cultured with cells derived from other embryonic tissues. **(A)** Schematic for experimental design to study cardiomyocyte differentiation of MSCs by co-culture with RECs. BM-MSCs were isolated from genetically modified mice that express α-MHC promoter-driven GFP. **(B)** RT-PCR primers were designed to specifically detect the expression of cardiac specific genes in mouse-derived mRNA (mHe) and do not amplify rat mRNA sequences (RECs). **(C)** Quantitative real time RT-PCR was performed to determine the expression of cardiac specific genes in mouse BM-MSCs after co-culture with RECs for 5 days. Data were normalized against the reference gene GAPDH and presented as relative units taking the expression in mouse heart as 1 unit. Data represent mean±SD of five independent experiments. *p<0.0001 between CC and MSCs groups derived from one-way ANOVA after Tukey's multiple comparisons test. **(D)** Representative images of BM-MSCs at 5 days of co-culture (original magnification 200x). MSCs were identified in the co-culture by Collagen type IV (Col IV) immunostaining. Cells undergoing cardiomyocyte differentiation express GFP (green cells). **(E)** α-MHC promoter activity was calculated as the percentage of Col IV-positive cells expressing GFP. Data represent mean±SD of four independent experiments. *p<0.0001 between groups derived from unpaired t test. **(F)** RT-PCR assay for the expression of cardiac specific genes in mice BM-MSCs after co-culture with rat embryonic cells from lung (RELu), kidney (REKi) and liver (RELi). The results of three independent co-culture experiments are shown. Abbreviations: α-MHC, alpha-myosin heavy chain; ANF, atrial natriuretic factor; CA, cardiac actin; CC, co-culture; Col IV, collagen type IV; GAPDH, glyceraldehyde 3-phosphate dehydrogenase; mHe, mouse heart; RECs, rat embryonic cardiomyocytes.

MSCs isolated from the BM of transgenic B-a-Fvb mice expressing GFP under the control of the cardiac specific α-myosin heavy chain promoter (α-MHC) were used for the co-culture experiments. As expected, GFP fluorescence was observed in the hearts of B-a-Fvb mice but not in other tissues, including BM (S1 Fig). In this way, we determined and identified the MSCs undergoing cardiomyocyte differentiation in the co-culture system (Fig 1A).

The acquisition of cardiac specific gene expression in BM-MSCs was assessed by using mouse specific primers to avoid the need to separate cells in the co-culture. According with this, the expression of Nkx2.5, atrial natriuretic factor (ANF) and α-cardiac actin (CA) was detected in mouse heart samples but not in RECs (Fig 1B). BM-MSCs only expressed these markers after 5 days of co-culture (Fig 1B). The expression of Nkx2.5 and ANF were significantly higher (7.40±0.78 and 5.73±0.51-fold, respectively) in co-cultured MSCs than in mouse heart (Fig 1C). The level of expression of CA in co-cultured MSCs was similar to that found in mouse heart (Fig 1C).

The frequency of MSCs differentiating towards the cardiomyocyte lineage was calculated by comparing the number of GFP+ cells (MSCs with an active α-MHC promoter) with the number of collagen type IV+ cells (MSCs in the co-culture) using immunocytochemistry (Fig 1D). The percentage of MSCs undergoing cardiomyocyte differentiation was 7.6±1.9% after 5 days of co-culture (Fig 1E), and was significantly higher than cell fusion events which occurred at a frequency of less than 1% in our co-culture system (S2 Fig).

When co-cultures were carried out with cells isolated from rat embryonic lung, kidney or liver tissue, BM-MSCs did not yield any GFP+ cells nor acquire the expression of cardiomyocyte specific genes (Fig 1F).

### OCT4 expression changes in MSCs after co-culture with RECs

**Fig 2:**
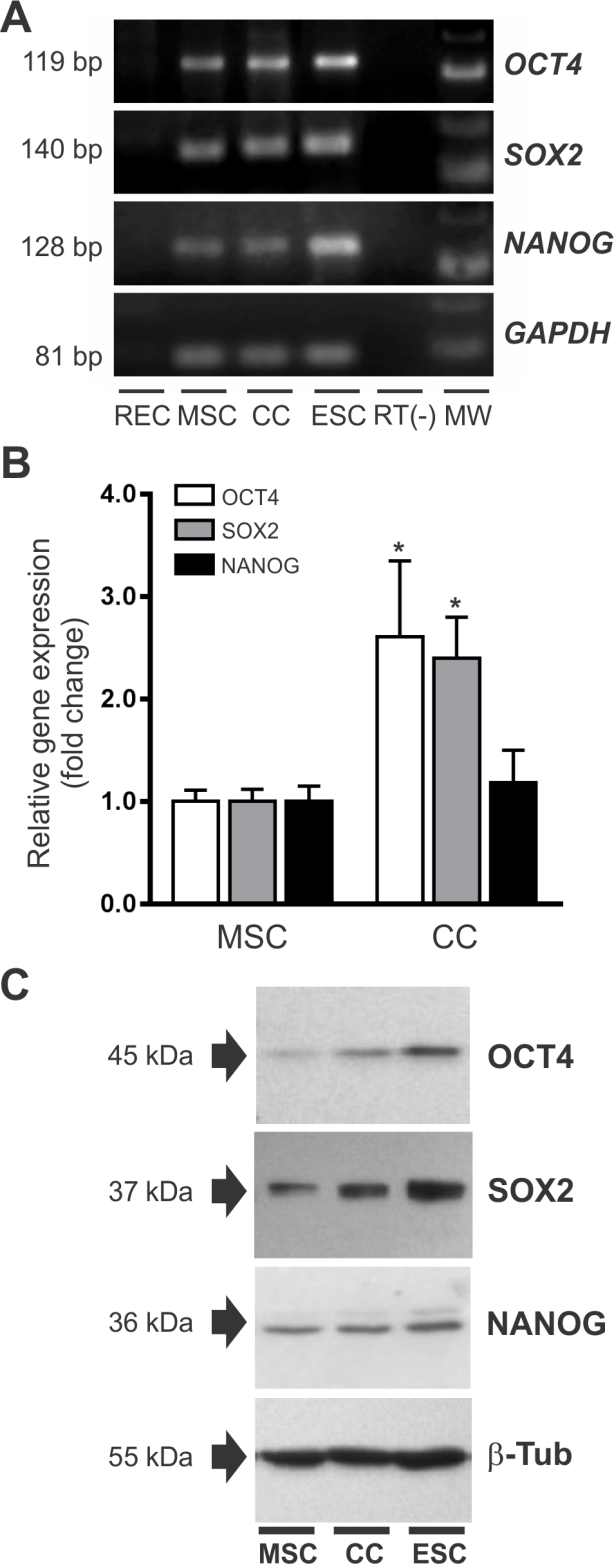
Changes in the expression of the pluripotency factors OCT4, SOX2 and NANOG in BM-MSCs after co-culture. **(A)** RT-PCR primers were designed to specifically detect gene expression of pluripotency factors in mouse mRNA (ESCs) without amplification of rat mRNA sequences (RECs). **(B)** Expression of pluripotency genes in untreated BM-MSCs and MSCs after co-culture with RECs as determined by qRT-PCR. Data were normalized against the reference gene GAPDH and presented as relative units taking the expression in untreated MSCs as 1 unit. Data represent mean±SD of five independent experiments. *p<0.001 between groups derived from unpaired t test. **(C)** Western blot showing changes in the expression of OCT4, SOX2 and NANOG in MSCs after the co-culture (n = 3). Total protein extract from ESC was used as positive control. Detection of β-Tub was used as loading control. Abbreviations: CC, co-culture; ESC, mouse embryonic stem cells; GAPDH, glyceraldehyde 3-phosphate dehydrogenase; MW, molecular weight marker; RECs, rat embryonic cardiomyocytes.

Changes in the expression of the pluripotency transcription factors OCT4, SOX2 and NANOG during the co-culture were evaluated by qRT-PCR analysis using mouse specific primers. As shown in Fig 2A, assay conditions allowed the detection of OCT4, SOX2 and NANOG gene expression in mice ESCs (positive control) but not in RECs. Interestingly, BM-MSCs basally expressed these stemness genes (Fig 2A). Immunocytochemistry analysis also showed that 83±9, 76±9 and 66±13% of BM-MSCs cultured under standard conditions were positive for the expression of OCT4, SOX2 and NANOG, respectively (S3 Fig). After 5 days of co-culture, the expression of OCT4 and SOX2 increased by 2.6±0.7- and 2.4±0.4-fold in MSCs, respectively, compared with baseline (Fig 2B). There was no significant difference in NANOG gene expression (Fig 2B). WB analysis showed augmented OCT4 and SOX2 protein expression in MSCs after co-culture, confirming qRT-PCR data (Fig 2C).

**Fig 3:**
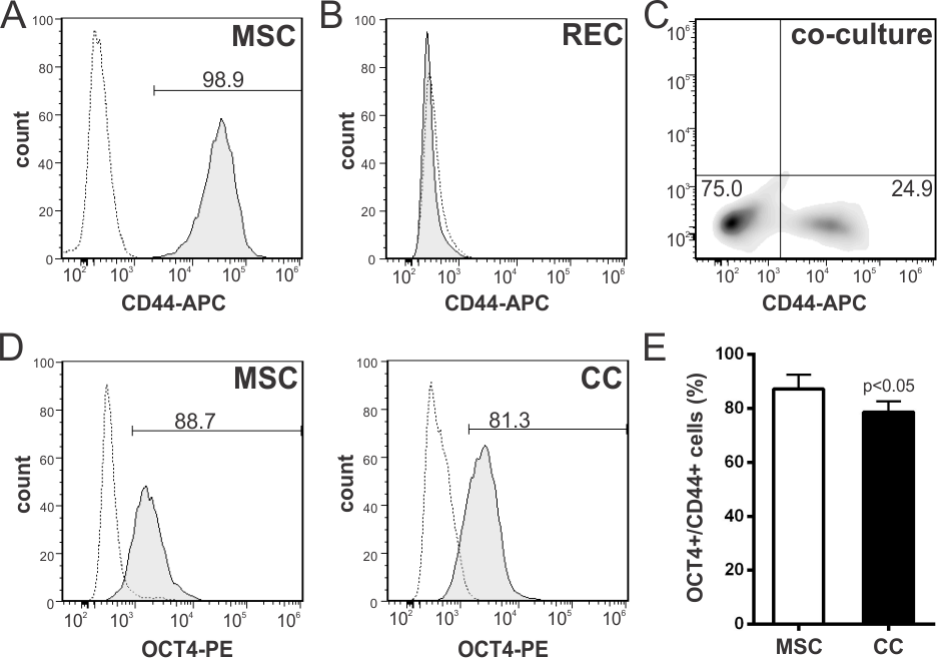
The frequency of OCT4 expression in BM-MSCs decreases after co-culture with RECs as assayed by flow cytometry. **(A)** BM-MSCs were stained with an APC-conjugated anti-mouse CD44 antibody. More than 98% of the cells are positive for this marker. Gray dotted line, isotype control; black line, antigen staining. **(B, C)** The CD44 antibody does not cross react with RECs and therefore was used to distinguish mouse from rat cells in the co-cultures. **(D)** OCT4 expression analyzed on the CD44+ gated population in untreated MSCs and MSCs after co-culture with RECs. Representative histograms. **(E)** Changes in the frequency of OCT4+ cells after the co-culture. Data represent mean±SD of four independent experiments. *p* value between groups derived from unpaired *t* test. Abbreviations: CC, co-culture; RECs, rat embryonic cardiomyocytes.

**Fig 4:**
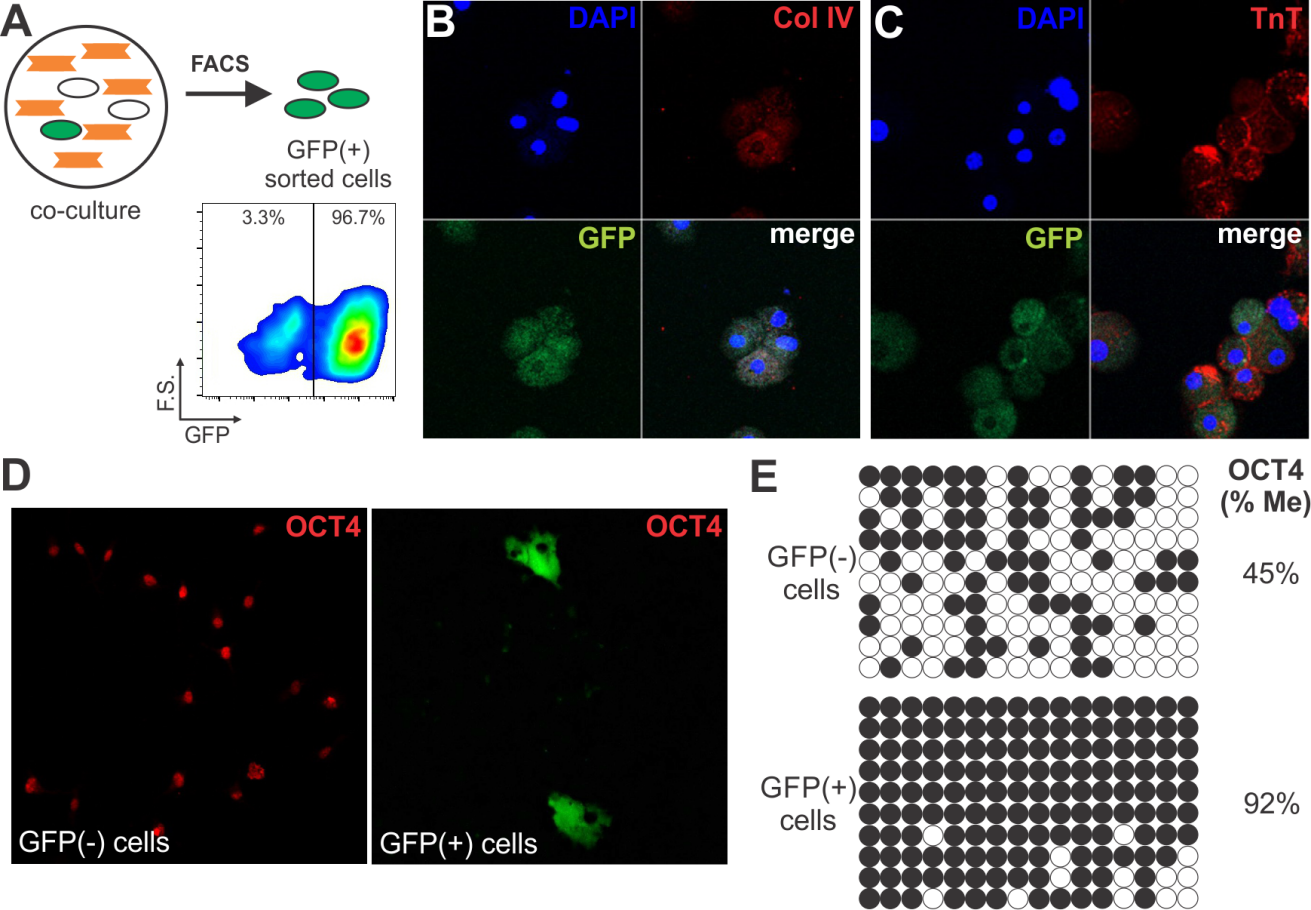
MSCs undergoing cardiomyocyte differentiation (GFP+ cells) lose expression of pluripotency factor, OCT4. **(A)** Schematic for GFP+ cell sorting after 5 days of co-culture with RECs. Representative dot plot illustrating forward-scatter vs GFP flow cytometry analysis on the GFP+/CD44+ gated population. **(B,C)** GFP+ sorted cells express the cardiac-specific protein troponin-T (TnT), but retained the expression of the stromal marker collagen type IV (Col IV). Images are representative of three independent experiments (original magnification 400x). **(D)** Immunocytochemical analysis of OCT4 expression in sorted GFP+ and GFP- MSCs after 5 days of co-culture. Images are representative of three independent experiments (original magnification 400x). **(E)** Bisulfite sequencing analysis of the OCT4 promoter in sorted GFP+ and GFP- MSCs after 5 days of co-culture. Each horizontal row of circles represents an individual sequencing reaction for a given amplicon. Open and closed circles indicate unmethylated and methylated CpGs, respectively. The overall percentage of methylation is noted to the right of each panel.

Of note, OCT4 expression increased in this differentiation model in contrast to its expected downregulation during the acquisition of a more differentiated phenotype [35]. However, both qRT-PCR and WB analyses were performed on the entire mixed population of MSCs. We therefore next determined the frequency of OCT4 expression in BM-MSCs before and after the co-culture using flow cytometry (Fig 3). To distinguish mouse from rat cells in co-culture, cells were stained with an anti-mouse CD44 antibody which does not cross react with rat CD44. CD44 was expressed on all MSCs and absent on RECs (Fig 3A and 3B). After 5 days of co-culture, the MSC population represented 20–25% of the total cell number, in agreement with the initial seeding ratio of 4 to 1 (RECs:MSCs) (Fig 3C). OCT4 expression was evaluated by gating on the CD44+ population. Flow cytometry analysis showed that 87±5% vs 79±4% (p<0.05) of MSCs expressed OCT4 at baseline and after the co-culture, respectively (Fig 3D and 3E). Interestingly, the frequency of MSCs that lost OCT4 (8%) was similar to the frequency of MSCs partially differentiating into cardiomyocytes (GFP+ cells) in our co-culture system, suggesting that MSCs undergoing differentiation may have lost OCT4. This notion is in agreement with the concept that OCT4 expression is repressed in differentiating cells. To test this hypothesis, we used cell sorting to separate and characterize the GFP+/CD44+ cell population (Fig 4). For all the experiments, we used a GFP+/CD44+ sorted population with a purity higher than 95% (Fig 4A). Immunocytochemistry analysis showed that GFP+/CD44+ cells co-expressed troponin T, a subunit of troponin which is essential for cardiac muscle contraction, and the stromal cell marker collagen type IV (Fig 4B and 4C). These results demonstrate the acquisition of at least one cardiac marker with the retention of a stromal determinant, inferring that the differentiation process is partial in our co-culture system. In addition, detection of DNA synthesis by BrdU incorporation assay showed that GFP+/CD44+ cells underwent cell cycle arrest (S4 Fig), a well-known characteristic of differentiating cardiomyocytes [36]. Notably, OCT4 was not expressed in MSCs undergoing cardiomyocyte differentiation (GFP+/CD44+), whereas undifferentiated cells (GFP-/CD44+) stained positive for nuclear OCT4 (Fig 4D). Current evidence suggests that DNA methylation is the major epigenetic mechanism regulating OCT4 expression [37]. The methylation profile of the OCT4 promoter was assessed by bisulfite DNA sequencing studying a 533 bp region that contains 16 CpG sites (S5 Fig). We found that the OCT4 promoter was hyper-methylated (>90%) in partially differentiated cells (GFP+/CD44+), while the methylation in undifferentiated MSCs was close to 50% (Fig 4E). This epigenetic mechanism may explain OCT4 repression in differentiating MSCs. Overall, these data demonstrate that there is an up-regulation of OCT4 expression in MSCs during the co-culture and that the cells undergoing partial cardiomyocyte differentiation lose expression of this factor. Our observation suggests that MSCs may undergo a de-differentiation process (gain in OCT4 expression) before being able to partially differentiate (S6 Fig). In this way, OCT4 modulates the interaction of MSCs with the microenvironment. Accordingly, when GFP-/CD44+ sorted cells were co-cultured with RECs again for other 5 days, the percentage of GFP+ cells (MSCs with an active α-MHC promoter) observed was 12.2±2.3%, indicating that there are temporal differences in the progression of differentiation among MSCs. In contrast, GFP+/CD44+ sorted cells cultured in complete media for 12 days, lose expression of GFP (inactive α-MHC promoter) and troponin T, while maintaining expression of Type IV collagen (S7 Fig). Most of the cells lose expression of GFP during the first 6 days in complete culture media followed by a significant rise in the proliferation rate. These results infer that the cardiac microenvironment is required to maintain the partial differentiated phenotype.

### OCT4 expression is required for partial cardiomyocyte differentiation of MSCs

**Fig 5:**
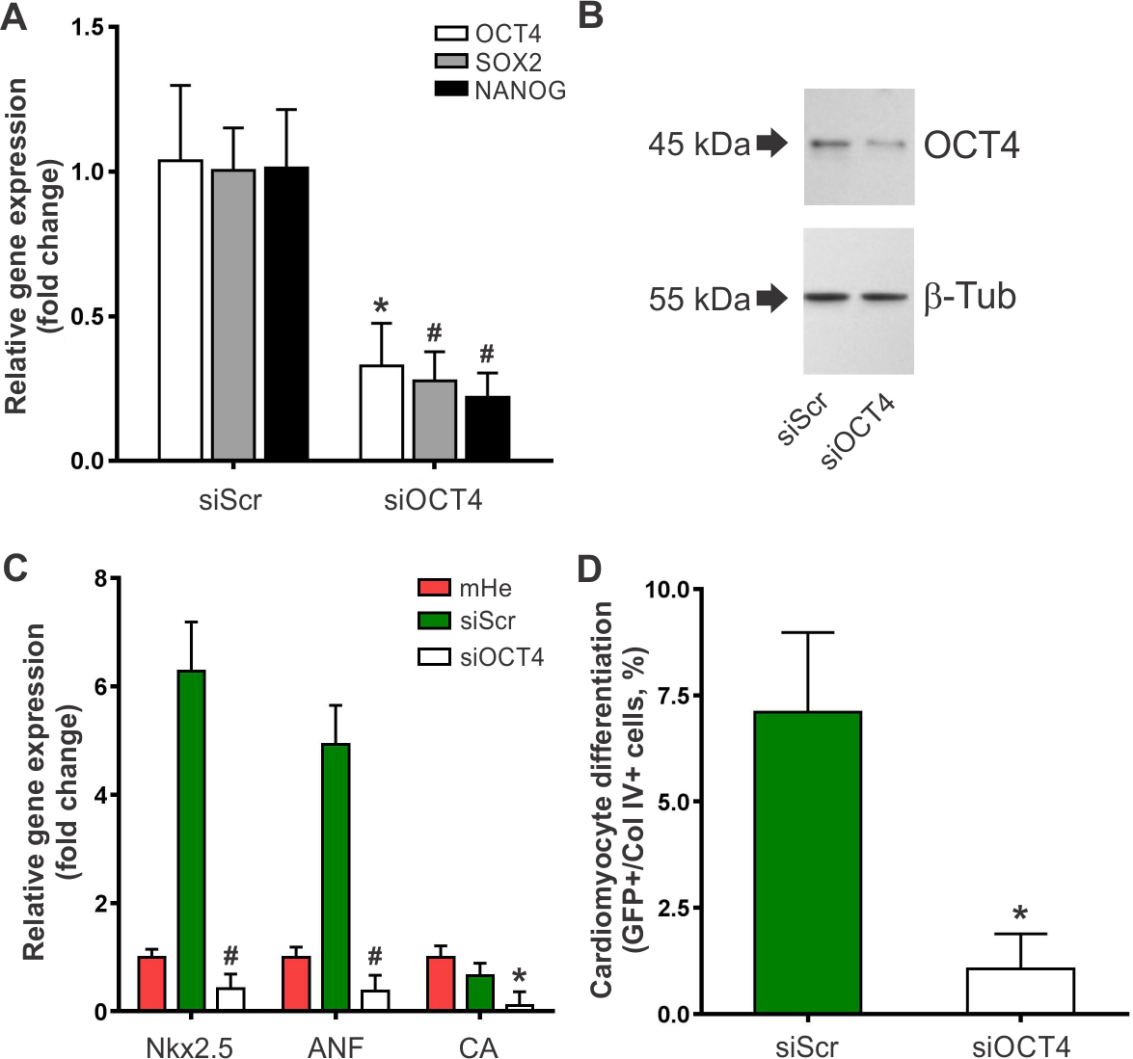
OCT4 expression is required for partial cardiomyocyte differentiation of MSCs during co-culture with RECs. BM-MSCs were incubated for 24 h with OCT4 siRNA (siOCT4) or a scrambled control siRNA (siScr) and transfected cells were then selected using puromycin. **(A)** Quantitative realtime PCR assay for expression of OCT4, SOX2 and NANOG in OCT4 siRNA silenced (siOCT4) and control siRNA MSCs (siScr). Individual PCR reactions were normalized against internal controls (GAPDH) and plotted relative to the expression level in untreated MSCs. Data represent mean±SD of four independent experiments. *p<0.01 and ^#^*p*<0.001 between groups derived from unpaired *t* test. **(B)** Western blot analysis of the OCT4 protein in OCT4 siRNA silenced (siOCT4) and control siRNA MSCs (siScr) (n = 3). **(C)** Expression of cardiac specific genes in siOCT4 vs. siScr MSCs after co-culture with RECs for 5 days as assessed by qRT-PCR. Data were normalized against the reference gene GAPDH and presented as relative units taking the expression in mouse heart as 1 unit. Data represent mean±SD of four independent experiments. *p<0.05 and ^#^*p*<0.0001 between siOCT4 and siScr MSCs groups derived from one-way ANOVA after Tukey's multiple comparisons test. **(D)** Cardiomyocyte differentiation frequency in siOCT4 vs. siScr MSCs after co-culture with RECs for 5 days. α-MHC promoter activity was calculated as the percentage of Col IV-positive cells expressing GFP. Data represent mean±SD of four independent experiments. *p<0.001 between groups derived from unpaired *t* test. Abbreviations: α-MHC, alpha-myosin heavy chain; ANF, atrial natriuretic factor; β-Tub, β-tubulin; CA, cardiac actin; CC, co-culture; Col IV, collagen type IV; GAPDH, glyceraldehyde 3-phosphate dehydrogenase; mHe, mouse heart; RECs, rat embryonic cardiomyocytes.

Finally, we studied whether OCT4 expression is directly related to the differentiation process. To this end, we carried out co-culture experiments employing an OCT4-silenced MSC population. We used a plasmid vector to drive high level expression of a hairpin siRNA against OCT4 (siOCT4). MSCs transfected with the siOCT4 plasmid showed a significant decrease in OCT4 expression compared with scrambled siRNA negative control (siScr) (Fig 5A and 5B). Interestingly, OCT4 silencing also decreased the expression of SOX2 and NANOG (Fig 5A), a result consistent with the role of OCT4 as an essential regulator of pluripotency. OCT4-silenced MSCs failed to express significant levels of cardiomyocyte genes when co-cultured with RECs for 5 days (Fig 5C). In addition, the frequency of differentiating cells (GFP+) significantly dropped to 1% (Fig 5D). These data infer that the interaction of MSCs with the cardiac microenvironment requires OCT4 expression to drive the cardiomyocyte differentiation process.

## Discussion

Cell therapy with MSCs holds promise for cardiac regeneration. While current evidence supports the MSC-mediated improvement of cardiac function by release of soluble growth factors and cytokines [13–16], the contribution of cardiomyocyte lineage commitment of MSCs at the injury site may also constitute a possible mechanism. In this regard, pre-conditioning of MSCs with different growth factors and cytokines before administration improves therapeutic efficacy [19,21]. We recently demonstrated that over 60% of donor cells lodging in the heart co-express cardiomyocyte and stromal markers in a mouse model of AMI [18]. Moreover, selective elimination of cardiovascular committed donor cells after transplantation of undifferentiated BM-MSCs decreases improvement in cardiac function [20]. These data infer that the interaction of MSCs with the cardiac niche is important to engender cardiac repair, even though the role of partially differentiated subpopulations is still poorly understood. The present study was designed to explore mechanisms mediating the differentiation of MSCs into cardiomyocytes using a co-culture system with RECs. Our results demonstrated that: i) MSCs acquire cardiac markers but retain the stromal phenotype when co-cultured with RECs; ii) this partial cardiomyocyte differentiation is directed by RECs but not by other types of embryonic cells; iii) OCT4 expression is required for the differentiation process; iv) de-differentiation is a central step in this mechanism.

We previously showed that the ability of MSCs to differentiate into functional cardiomyocytes does not occur *in vitro* [17,22]. The cells of MSC origin exhibiting cardiac-specific gene expression neither generated spontaneous or evoked action potentials nor produced ionic currents typical of cardiomyocytes [17]. Other studies have also concluded that MSCs do not differentiate into functional cardiomyocytes [38,39]. The extent of this differentiation, however, is similar to that acquired by most donor cells *in vivo* [18]. Here we found that co-culture with other embryonic cells failed to induce MSC cardiomyocyte differentiation, indicating that unspecified embryonic factors are unlikely to mediate this process. Thus, our co-culture with RECs provides a suitable *in vitro* model to identify molecules involved in the interaction of MSCs with the cardiac microenvironment. Labile short distance factors released by RECs may play a major role in directing MSC cardiomyocyte reprogramming as the frequency and extent of differentiation achieved using transwell membranes was similar to cell co-culture [22]. The identification of the specific mechanisms mediating this process remains elusive as they may include not only soluble cytokines and growth factors but also extracellular vesicles and intercellular transfer of mitochondria.

**Fig 6:**
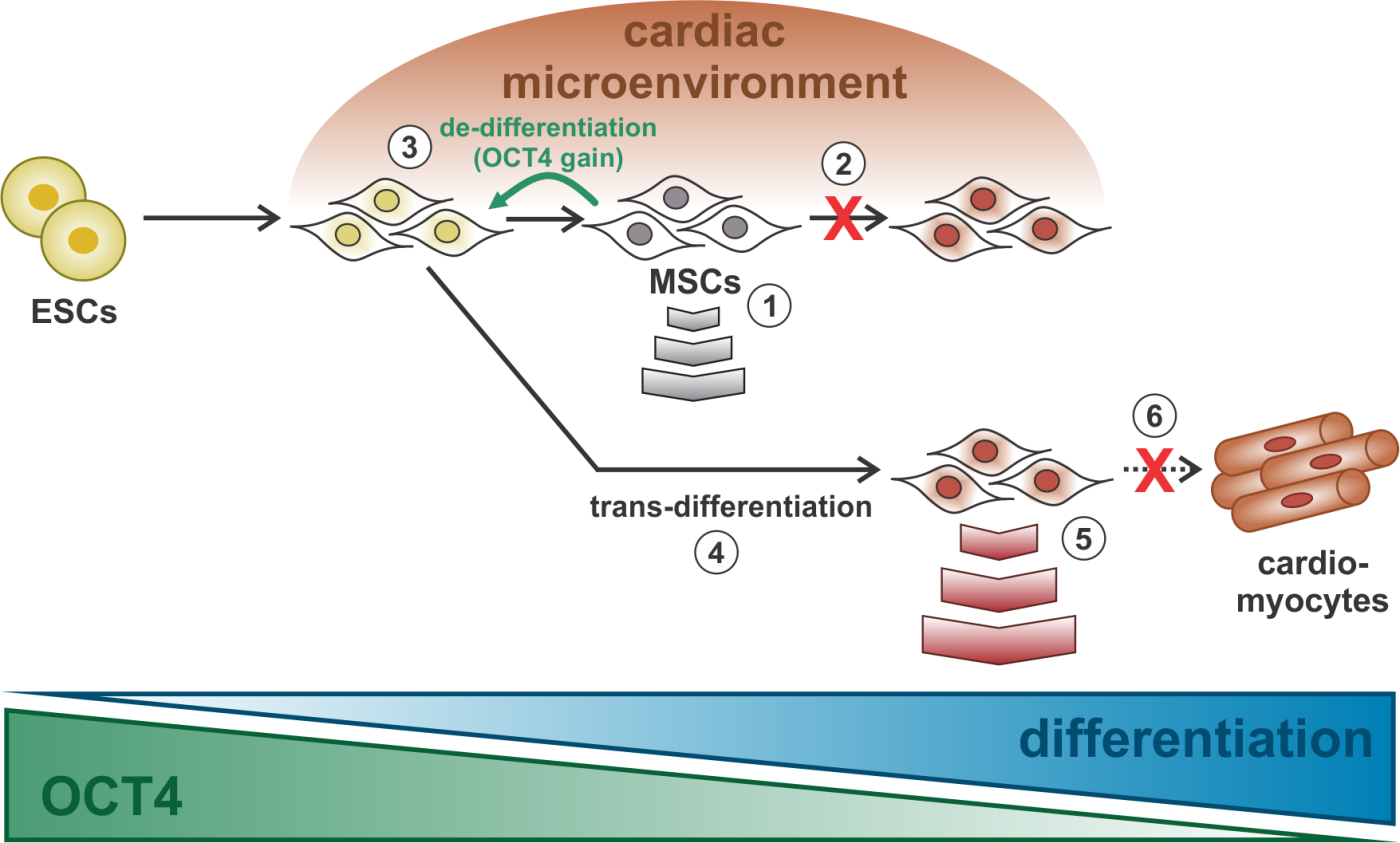
OCT4 expression is required for interaction of MSCs with the cardiac microenvironment. A schematic depicting the main mechanisms proposed in the present study. **(1)** MSCs have a basal expression of OCT4 and release several growth factors and cytokines under normal conditions. **(2)** MSCs interact with the cardiac microenvironment and partially differentiate into cardiomyocytes, by an indirect mechanism. **(3)** As a result of the interaction with the cardiac microenvironment, MSCs de-differentiate with a net gain in OCT4 expression. **(4)** De-differentiated MSCs express higher levels of OCT4 and can start the differentiation process into cardiomyocytes. **(5)** MSCs acquire a partial cardiomyocyte phenotype that modulates the paracrine effect and improves their cardiac regenerative potential. **(6)** Full cardiomyocyte differentiation of MSCs to generate completely mature cardiomyocytes was not observed. Abbreviations: ESC, embryonic stem cells; MSCs, mesenchymal stromal cells.

Previous reports have shown that MSCs express low levels of OCT4 at early passage and suggested that this pluripotency factor could be a marker of MSC differentiation potential [28,40]. Differences in the multipotency of MSCs isolated from different sources can be attributed to relatively slight changes in OCT4 expression, as the multipotency of MSCs may be epigenetically restricted [23]. The loss of proliferation and differentiation potential with increasing donor age may also be related to limited OCT4 expression, as it occurs with extended passaging *in vitro* [29,41]. In contrast, ectopic expression of OCT4 enhances the adipogenic and osteogenic differentiation of MSCs [42,43]. Moreover, OCT4 overexpression in human amnion mesenchymal cells improves their differentiation potential into cardiomyocytes [44]. In view of these data, it has been suggested that forced expression of OCT4 favors the maintenance of MSCs in the mesodermal lineage [43]. Whether this occurs under more physiological conditions is uncertain as ectopic OCT4 expression usually increases more than 100-fold [43,44]. Interestingly, we found that OCT4 expression increased by 2.6-fold in MSCs during the co-culture with RECs. This up-regulation arising from the interaction of MSCs and the cardiac microenvironment may mediate cardiomyocyte reprogramming. In fact, a two-fold increase in OCT4 expression was associated with differentiation of ESCs into primitive endoderm and mesoderm [45]. After 5 days in co-culture, up to 8% of the MSCs acquired expression of cardiac markers. Of note, the frequency of OCT4+ MSCs in the co-culture decreased by a similar percentage. We further demonstrated that these partially differentiated cells lost OCT4 expression and proliferation potential. It is well-known that during differentiation, cells exhibit little proliferative activity. ESCs differentiate into a specific lineage by losing expression of pluripotent genes accompanied by the gradual activation of differentiation genes [35]. Moreover, increased DNA methylation of pluripotency-associated genes has been observed during ESCs differentiation, suggesting that this mechanism is important for repressing pluripotency [37,46]. The same mechanism may also operate in MSCs. We found that the OCT4 promoter was hypermethylated in MSCs undergoing partial cardiomyocyte differentiation (GFP+/CD44+ cells). Nonetheless, these cells lose the expression of cardiac markers and start to proliferate when cultured in complete culture media, supporting the notion that this partial cardiomyocyte phenotype is maintained by an interaction with the cardiac microenvironment and may be analogous to the stem cell niche determining the fate of stem cells [47]. Specific signals from the microenvironment influence stem cell identity and may promote differentiation. The observation that MSCs acquired a partial cardiomyocyte phenotype under co-culture conditions which was lost upon withdrawal of cardiac microenvironmental stimuli, suggests that MSCs can return to their original identity. Partially differentiated cells may be more competent to revert to the starting fate than cells that have undergone stable differentiation [48]. We had previously proposed a link between partial cardiomyocyte differentiation and paracrine effects [22]. Thus, the release of soluble growth factors and cytokines may be influenced by the interaction of MSCs with the local environment to improve therapeutic potency (Fig 6).

Transdifferentiation was initially defined as the conversion of one differentiated cell type into another [49]. This change in differentiation at the cellular level may occur either directly, without any reversion to immature phenotypes, or through a de-differentiation step before cell redifferentiation to a new mature phenotype [48]. Previous studies showed direct conversion of differentiated cells from one lineage into another, such as the conversion of fibroblasts to multilineage blood progenitors, functional cardiomyocytes or neurons [50–52]. In all cases, the direct conversion involves a significant genomic reorganization achieved by forced differentiation through the expression of ectopic factors. MSCs were able to directly switch from osteogenically differentiated cells into adipogenically or chondrogenically differentiated cells showing true differentiation potential, as controversies such as progenitor cell contamination, cell fusion and other cellular artifacts were excluded by tracking cells with GFP [53]. More recently however, Ullah et al. showed by single cell analysis that differentiation of MSC-derived adipocytes into osteogenically or chondrogenically differentiated cells occurs via de-differentiation [54]. Attempts to directly convert differentiated cells from one lineage to another resulted in a mixed culture, in which only some cells were able to differentiate. Moreover, single cell analysis ruled out direct cell-to-cell conversion and supported de-differentiation as an intermediate step during transdifferentiation [54]. Similarly, it has been reported that de-differentiation is a necessary step for switching bone marrow-derived neurons to epithelial cells or vice versa [55]. Nevertheless, a deeper mechanistic understanding of MSC genetic reprogramming is needed. A previous study demonstrated that MSC-derived neurons can revert to a stromal phenotype upon withdrawal of extrinsic stimulation [56]. Moreover, these de-differentiated MSCs may represent neural stem/progenitor cells as they exhibited enhanced cell survival and higher efficacy in neuronal differentiation compared with naïve MSCs [56]. More recently, the same was established after induction of osteogenic differentiation. De-differentiated osteogenic MSCs showed enhanced differentiation along the osteoblastic lineage, as well as increased cell survival and colony forming ability [57]. These cells also gained specific epigenetic changes with enhanced expression of OCT4, SOX2 and NANOG [57]. Of note, our study revealed that global OCT4 expression increased in MSCs after 5 days in co-culture while cells undergoing cardiomyocyte differentiation lost OCT4. It is logical to infer a net gain in OCT4 expression in undifferentiated MSCs. This undifferentiated population (GFP-/CD44+), however, was able to generate partially differentiated cardiomyocytes when co-cultured again for another 5 days. These data suggest that partial cardiomyocyte differentiation of MSCs involves a de-differentiation intermediate with increased OCT4 expression. For MSCs, a small minority of which comprise adult stem cells, de-differentiation may represent a relatively small step backwards. Differences in the timing of differentiation may be related to cell heterogeneity among the MSCs. For example, a subpopulation of early progenitor cells with enhanced expression of OCT4 present at early passage can be selected by serum deprivation [40]. This subpopulation may differentiate more easily and faster than other MSCs with reduced OCT4 levels. The latter may require a greater change to revert to a more progenitor-like cell. In both cases, the differentiation occurs in a stepwise fashion, resembling genetic reprogramming to pluripotency. Consequently, the plasticity of MSCs depends on their ability to de-differentiate to more primitive stem cells, which can then be reprogrammed to differentiate along another lineage in response to environmental signals. OCT4 may play a key role in this process. Ectopic expression of OCT4 in certain somatic cells has been associated with active de-differentiation [58]. We found that blocking OCT4 expression by siRNA significantly reduced the ability of MSCs to differentiate in our co-culture system. Our data suggest that OCT4 up-regulation is required and supports the notion that de-differentiation is an integral part of partial cardiomyocyte differentiation of MSCs (Fig 6).

## Conclusions

We showed that OCT4 expression mediates the partial cardiomyocyte differentiation of BM-MSCs upon co-culture with RECs. We found that activation of pluripotent genes in MSCs during co-culture is required, inferring that the differentiation process proceeds via de-differentiation-mediated reprogramming. De-differentiation redirects cell fate by reverting differentiated cells to an earlier, more primitive phenotype with extended differentiation potential. Thus, it is not surprising that a master regulator of pluripotency such as OCT4 may control this process. Other studies have shown that MSCs are able to differentiate after successive changes in induction media. It is noteworthy that our co-culture system resembles the cardiac microenvironment, hence our data represent the response of MSCs in a more physiological situation. Moreover, here we provide an OCT4-dependent mechanism that explains our previous data supporting the view that partial cardiomyocyte reprograming may be important to engender cardiac repair.

## Supporting information

S1 Text Additional materials and methods. (PDF)

S1 Table DNA primer sequences. (PDF)

S1 Fig Green fluorescent protein (GFP) expression in B-a-Fvb transgenic mice. **(A)** Expression of GFP is driven by the α-myosin heavy chain promoter. **(B)** Bright field and fluorescence images of B-a-Fvb (left) and wildtype (right) hearts showing the expression of GFP in the myocardium of B-a-Fvb mice. **(C)** Northern blot analysis confirming that GFP is only expressed in the hearts of the B-a-Fvb transgenic mice. No GFP expression was detected in wildtype hearts or the other indicated tissues of the B-a-Fvb mice.
(TIF)

S2 Fig Determination of cell fusion events. For cell fusion studies, co-culture experiments were done as described in Materials and Methods, except that RECs were only obtained from male embryos and BM-MSCs from female mice. After 5 days of co-culture, cells were fixed and subjected to fluorescence in situ hybridization (FISH) for X and Y chromosomes. The probe for detection of Y chromosomes was rat specific (rat Y chromosome-Cy5, Cambio, UK). The probe for detection of X chromosomes was mouse specific (mouse X chromosome-Cy3, Cambio, UK). Visualization of cells with two X chromosomes (from female mice) and one Y chromosome (from male rats) were taken to have undergone a fusion event (XXY chromosomes). Nuclei were stained using 4,6-diamidino-2-phenylindole (DAPI), which was used to quantify cell numbers.
(TIF)

S3 Fig OCT4, SOX2 and NANOG expression in BM-MSCs by immunocytochemistry. Immunofluorescence staining of the pluripotency markers OCT4, SOX2 and NANOG in BM-MSCs (original magnification 200x). Nuclei were stained with DAPI to quantify cell numbers.
(TIF)

S4 Fig Partial cardiomyocyte differentiated-MSCs (GFP+ cells) undergo cell cycle arrest. BrdU incorporation assay was performed to detect DNA synthesis in BM-MSCs in the co-culture system. BM-MSCs isolated from GFP-Balb/c mice were used as an intrinsically labelled GFP control. After 5 days of co-culture, proliferating cells were marked with BrdU and analyzed by immunofluorescence as described above. Negative control: GFP-Balb/c MSCs after the co-culture but without BrdU staining. Positive control: GFP-Balb/c MSCs after the co-culture with BrdU staining (BrdU+/GFP+ cells represents the total percentage of MSCs proliferating after 5 days in co-culture). When MSCs derived from b-a-FvB mice were used for the co-culture experiments, GFP+ cells represent the MSCs undergoing partial cardiomyocyte differentiation (α-MHC active promoter). Data represent mean±SD of four independent experiments.
(TIF)

S5 Fig Bisulfite genome sequencing. **(A)** Mouse OCT4 promoter sequence was analyzed by MethPrimer software. CpG methylation sites are individually shown in red. The OCT4 promoter region studied is encompassed by the green arrows (inner primers). **(B)** Nucleotide sequence of the OCT4 promoter region analyzed by bisulfite DNA sequencing. The 533 bp region starts approximately 500bp upstream of the transcription initiation site and contains 16 CpG sites. Different elements are highlighted in colors: green, specific primer sequences; red, CpG methylation sites; purple, open reading frame.
(TIF)

S6 Fig Schematic diagram representing the changes in OCT4 expression during partial cardiomyocyte differentiation of MSCs. MSCs constitute a heterogeneous population of cells with a small range of OCT4 expression, which is related to their proliferation and multipotency capacity. Upon co-culture with REC, MSCs de-differentiate with a gain in OCT4 expression before being able to partially transdifferentiate into cardiomyocytes. MSCs starting with a high level of OCT4 expression completes this process within 5 days of co-culture, whereas de-differentiation takes longer for MSCs with low OCT4. Consequently, differences in the timing of reprogramming into cardiomyocytes may be due to cell heterogeneity among the MSCs.
(TIF)

S7 Fig GFP+ sorted cells lose the expression of GFP and cardiac troponin-T (TnT) when culture in complete culture media. **(A, B)** GFP+ sorted cells express the stromal marker collagen type IV (Col IV) but lose the expression of the cardiac-specific protein troponin-T (TnT) after 12 days of culture in complete culture media. Images are representative of three independent experiments. **(C)** Growth curve and GFP expression on GFP+ sorted cells cultured under conventional conditions. Data represent mean±SD of three independent experiments.
(TIF)
