# Supplementary material for: OCT4 expression mediates partial cardiomyocyte reprogramming of mesenchymal stromal cells
Source: PLoS One. 2017 Dec 7;12(12):e0189131. doi: 10.1371/journal.pone.0189131 (PMC5720736; doi:10.1371/journal.pone.0189131)
Supplement: S1 Text — (PDF) [file pone.0189131.s001.pdf]

S1 Text. Additional Materials and Methods

RT-PCR analysis

Total RNA was extracted from cells using Trizol reagent (Invitrogen) as described by the manufacturer. The amount and quality of RNA isolated was determined by measuring the absorbance at 260 nm and the 260/280 ratio using a Nanodrop spectrophotometer (Thermo Fisher Scientific). One microgram was reverse transcribed into cDNA using random primers and MultiScribe RT following the manufacturer instructions (High-Capacity cDNA Reverse Transcription Kit, Applied Biosystems). A PCR reaction mixture (25 μl) containing cDNA preparation (1 μl), 1 x PCR buffer, 1.5 mM $MgCl_2$, 200 μM dNTPs, primers (0.2 μM each) and 0.625 U Taq DNA polymerase (Invitrogen) was used. Cycling conditions were as follows: 94°C for 1 min, then 30 cycles at 94°C denaturing for 15 s, 55°C annealing for 30 s, and 72°C extension for 30 s, and a final step of 72°C for 2 min. The amplicons were visualized on 1.5% agarose gels with the use of ethidium bromide. Specific amplification products of the expected size were observed and their identities were confirmed by automatic DNA sequencing. Ethidium bromide stained gels were photographed using a Bio-Rad Molecular Imager Gel Doc XR+ System (Bio-Rad Laboratories).

Immunocytochemistry

To determine the basal expression of OCT4, SOX2 and NANOG in BM-MSCs, cells in primary cultures were fixed in 4% paraformaldehyde/PBS for 10 min, permeabilized with 0.2% Triton X-100/PBS for 10 min, and then blocked with 5% FBS/PBS for 1 h. Cells were then incubated overnight at 4°C with primary antibodies against OCT4 (1:250), SOX2 (1:250), and NANOG (1:200). After incubation, cells were washed and incubated for 1 h at room temperature with the secondary antibody Alexa Fluor 594-conjugated donkey anti-rabbit IgG (1:250, A-21207, Molecular Probes). Nuclei were stained with DAPI (DAPI ProLong Gold, Invitrogen), which was used to quantify cell numbers. Detection of fluorescent signals was performed using confocal laser scanning microscopy (Fluoview FV1000, Olympus, Tokyo, Japan).

BrdU incorporation assay

BrdU incorporation was used to evaluate whether cell cycle arrest occurs in BM-MSCs undergoing partial cardiomyocyte differentiation (GFP+ cells). Co-culture experiments were carried out using b-a-Fvb-derived BM-MSCs and REC as described in Materials and Methods. We also employed an intrinsically GFP-marked stromal cell population isolated from GFP-Balb/c mice as control. After 5 days of co-culture, cells were re-incubated with 10 μM BrdU (Thermo Fisher Scientific, B23151) for 6 h at 37°C. After removing the culture media, cells were fixed in methanol:acetone (1:1), DNA denatured by an acid treatment (2N HCl), and permeabilized in 0.2% Triton X-100 in PBS. Immunofluorescence was carried out using antibodies against BrdU (1:1000, Thermo Fisher Scientific, MA3-071) followed by staining with Alexa Fluor 594 goat anti-mouse (Thermo Fisher Scientific, A-11032). To visualize all nuclei, DNA was stained with DAPI. Percentage of BrdU-positive cells was determined from the ratio of BrdU-positive nuclei over total DAPI-stained nuclei. Detection of fluorescent signals was performed using confocal laser scanning microscopy (Fluoview FV1000, Olympus, Tokyo, Japan).
