# Supplementary material for: OCT4 expression mediates partial cardiomyocyte reprogramming of mesenchymal stromal cells
Source: PLoS One. 2017 Dec 7;12(12):e0189131. doi: 10.1371/journal.pone.0189131 (PMC5720736; doi:10.1371/journal.pone.0189131)
Supplement: S1 Table — (PDF) [file pone.0189131.s002.pdf]

Supplementary Table 1. DNA primer sequences.

Primer name	Sequence
Gene expression	
Nkx2.5 forward[1]	ATGCCTATGGCTACAACGC
Nkx2.5 reverse[1]	ACTCACTTTAATGGGAAGAGGG
ANF forward[1]	TTGGCTTCCAGGCCATAATTG
ANF reverse[1]	AAGAGGGCAGATCTATCGGA
α-CA forward[1]	TGTTACGTCGCCTTGGATTTTGAG
α-CA reverse[1]	AAGAGAGAGACATATCAGAAGC
OCT4 forward	CAATGCCGTGAAGTTGGAG
OCT4 reverse	CCAAGGTGATCCTCTTCTGC
SOX2 forward	ACATGTGAGGGCTGGACTG
SOX2 reverse	CGTTTCGCTGCGGAGAT
NANOG forward	TCTGGGAACGCCTCATCA
NANOG reverse	AGAGGCAGGTCTTCAGAGGAA
GAPDH forward	ATGGTGAAGGTCGGTGTGA
GAPDH reverse	CTCCACTTTGCCACTGCAA
Bisulfite sequencing[2]	
OCT4-out forward	GAGGATTGGAGGTGTAATGGTTGTT
OCT4-out reverse	CTACTAACCCATCACCCCCACCTA
OCT4-in forward	TGGGTTGAAATATTGGGTTTATTT
OCT4-in reverse	CTAAAACCAAATATCCAACCATA

[1] Rose et al., Stem Cells 2008;26(11):2884-92.

[2] Gao et al., Cell Stem Cell 2013;12(4):453-69.
