# Supplementary figures and images for: OCT4 expression mediates partial cardiomyocyte reprogramming of mesenchymal stromal cells
Source: PLoS One. 2017 Dec 7;12(12):e0189131. doi: 10.1371/journal.pone.0189131 (PMC5720736; doi:10.1371/journal.pone.0189131)

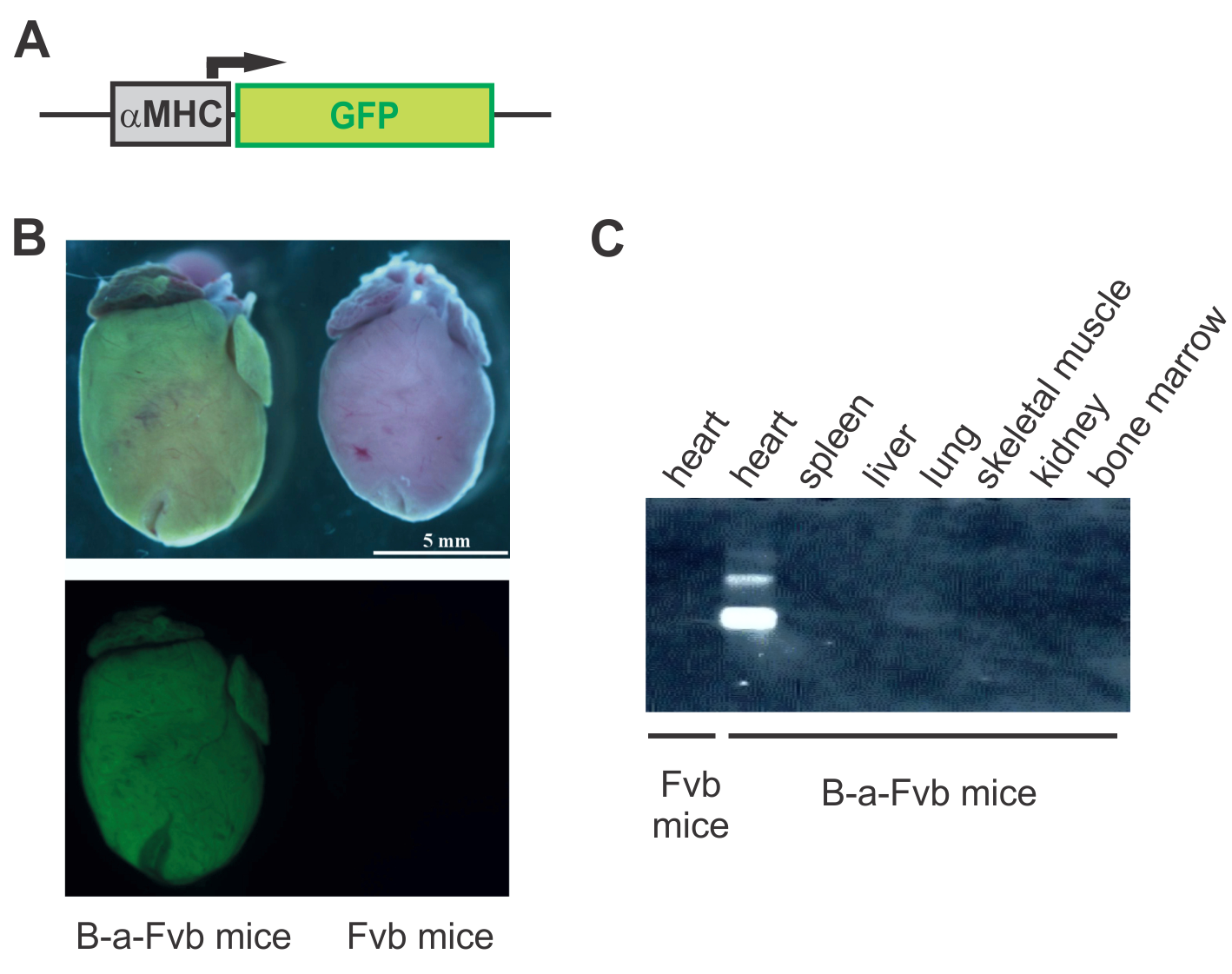

Supplement: S1 Fig — (A) Expression of GFP is driven by the α-myosin heavy chain promoter. (B) Bright field and fluorescence images of B-a-Fvb (left) and wildtype (right) hearts showing the expression of GFP in the myocardium of B-a-Fvb mice. (C) Northern blot analysis confirming that GFP is only expressed in the hearts of the B-a-Fvb transgenic mice. No GFP expression was detected in wildtype hearts or the other indicated tissues of the B-a-Fvb mice. (TIF) [file pone.0189131.s003.tif]

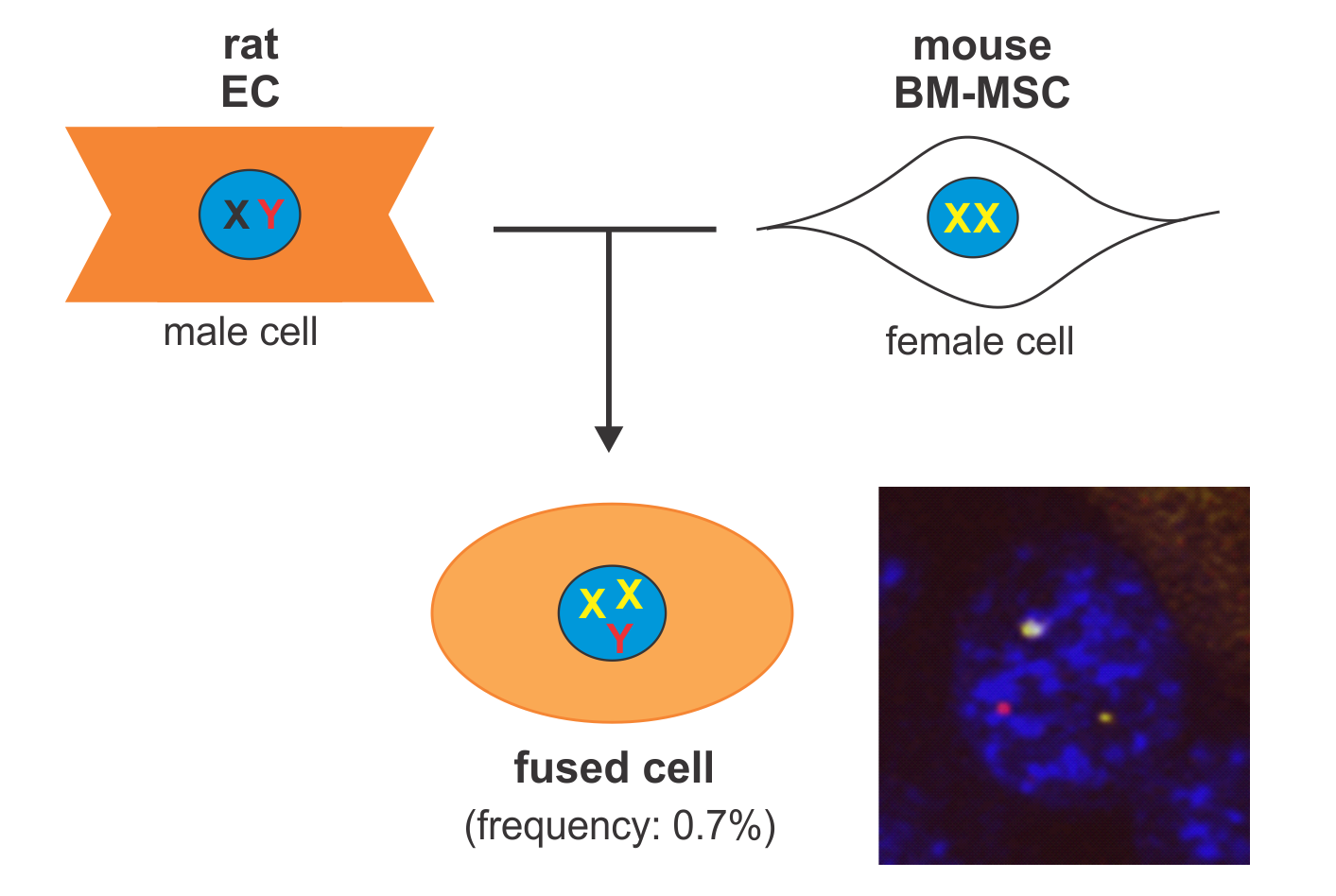

Supplement: S2 Fig — For cell fusion studies, co-culture experiments were done as described in Materials and Methods, except that RECs were only obtained from male embryos and BM-MSCs from female mice. After 5 days of co-culture, cells were fixed and subjected to fluorescence in situ hybridization (FISH) for X and Y chromosomes. The probe for detection of Y chromosomes was rat specific (rat Y chromosome-Cy5, Cambio, UK). The probe for detection of X chromosomes was mouse specific (mouse X chromosome-Cy3, Cambio, UK). Visualization of cells with two X chromosomes (from female mice) and one Y chromosome (from male rats) were taken to have undergone a fusion event (XXY chromosomes). Nuclei were stained using 4,6-diamidino-2-phenylindole (DAPI), which was used to quantify cell numbers. (TIF) [file pone.0189131.s004.tif]

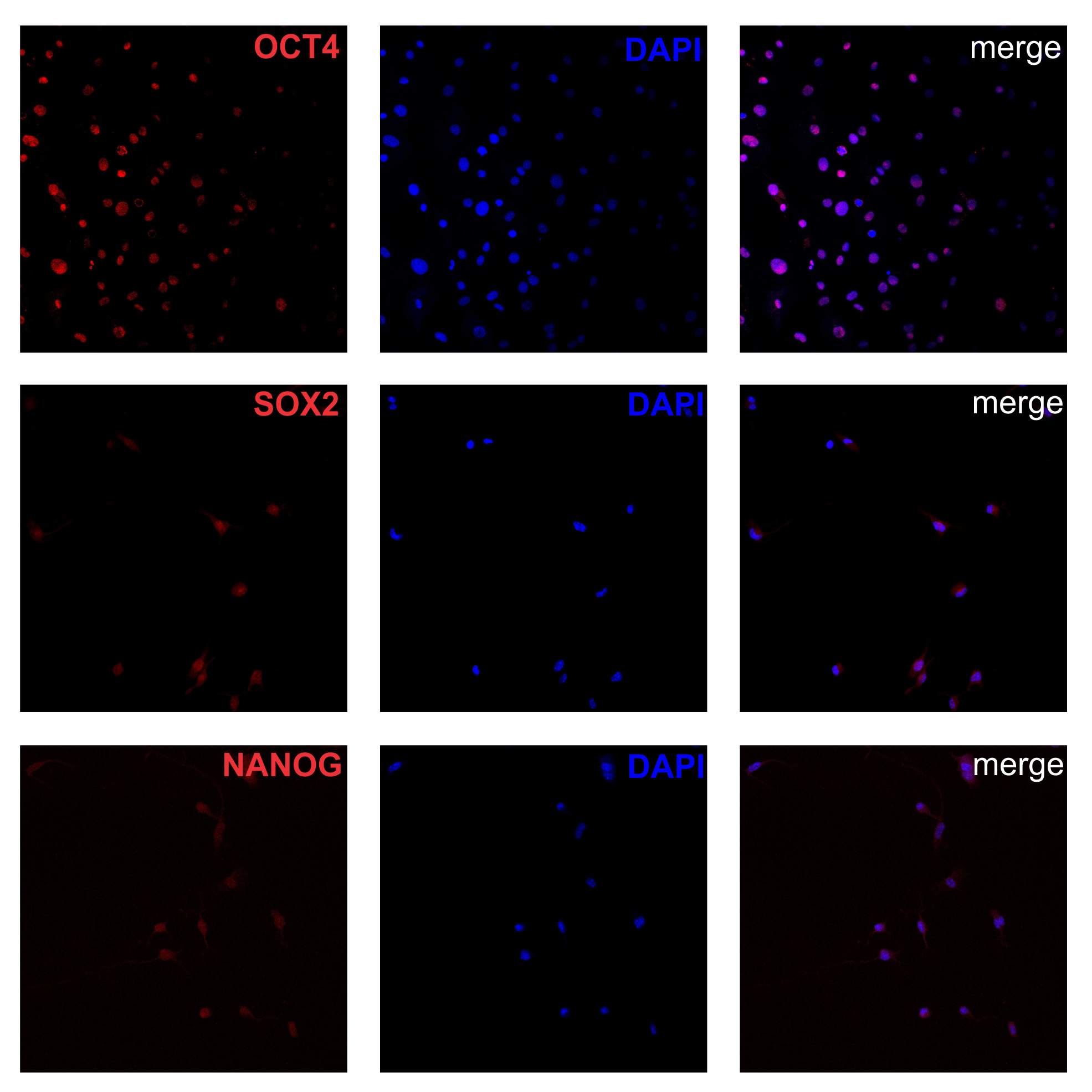

Supplement: S3 Fig — Immunofluorescence staining of the pluripotency markers OCT4, SOX2 and NANOG in BM-MSCs (original magnification 200x). Nuclei were stained with DAPI to quantify cell numbers. (TIF) [file pone.0189131.s005.tif]

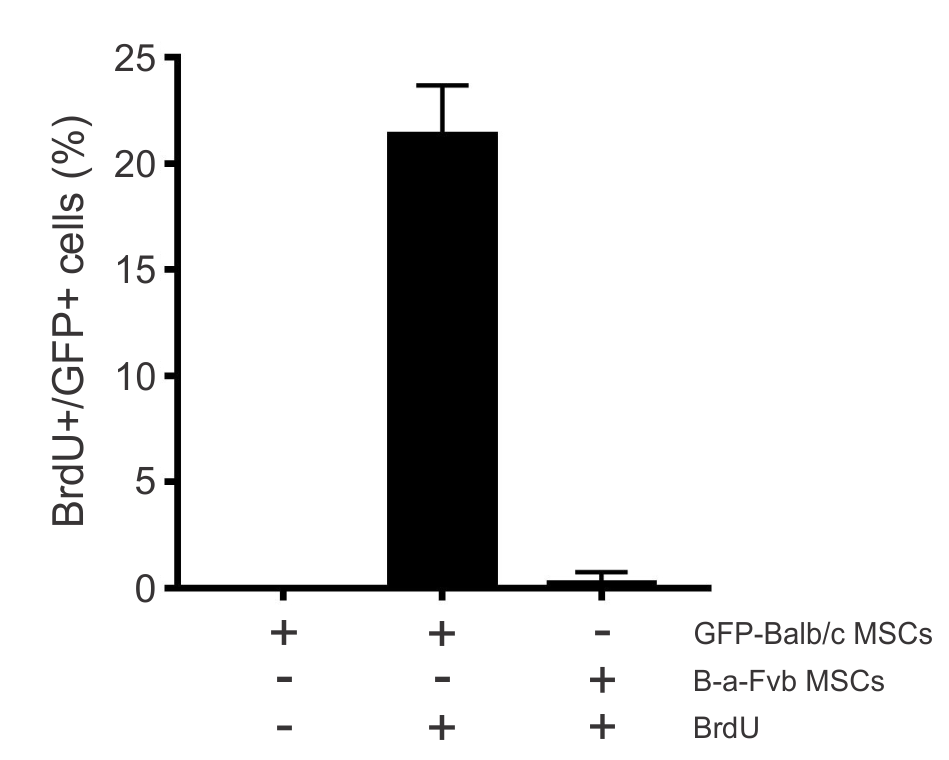

Supplement: S4 Fig — BrdU incorporation assay was performed to detect DNA synthesis in BM-MSCs in the co-culture system. BM-MSCs isolated from GFP-Balb/c mice were used as an intrinsically labelled GFP control. After 5 days of co-culture, proliferating cells were marked with BrdU and analyzed by immunofluorescence as described above. Negative control: GFP-Balb/c MSCs after the co-culture but without BrdU staining. Positive control: GFP-Balb/c MSCs after the co-culture with BrdU staining (BrdU+/GFP+ cells represents the total percentage of MSCs proliferating after 5 days in co-culture). When MSCs derived from b-a-FvB mice were used for the co-culture experiments, GFP+ cells represent the MSCs undergoing partial cardiomyocyte differentiation (α-MHC active promoter). Data represent mean±SD of four independent experiments. (TIF) [file pone.0189131.s006.tif]

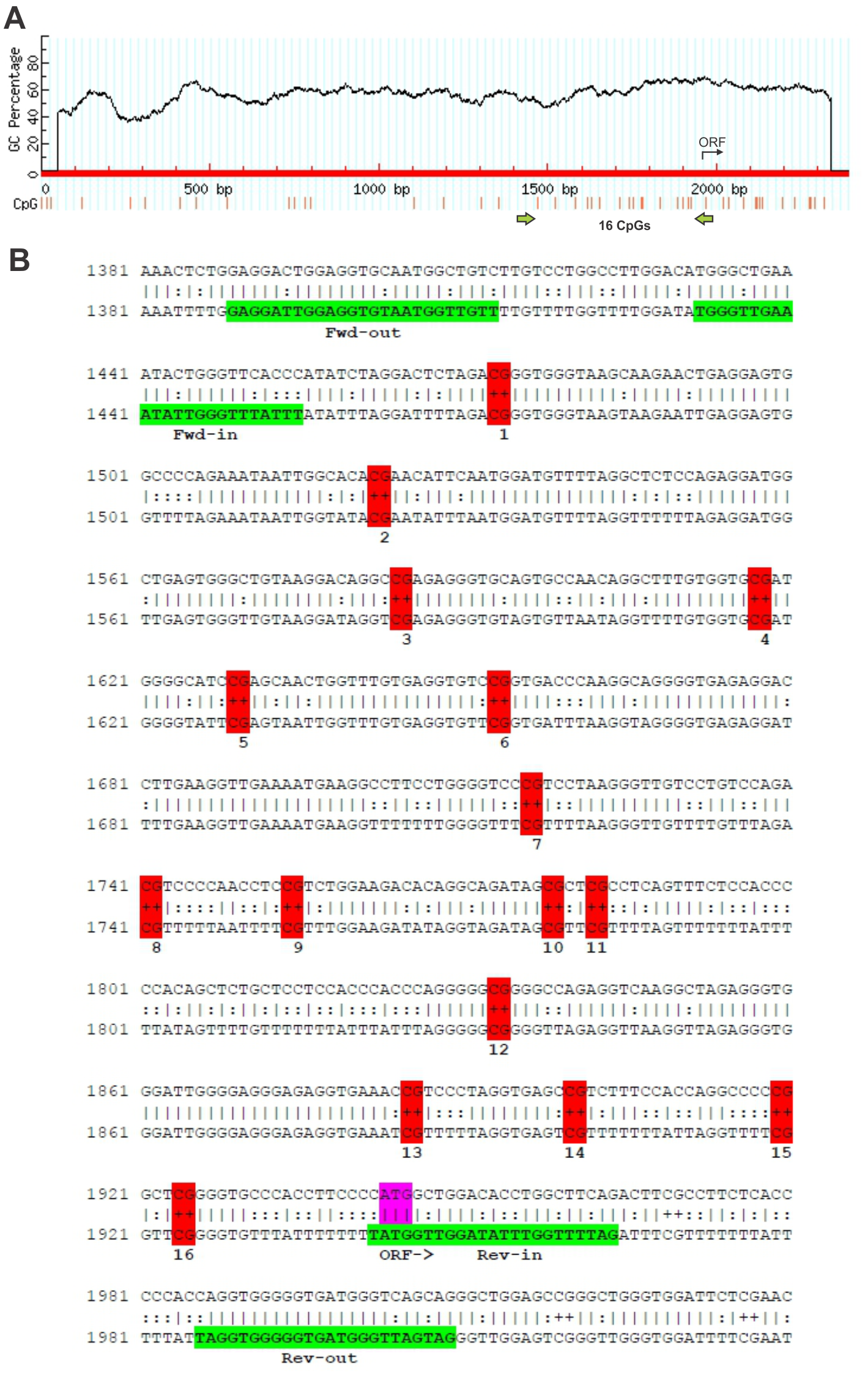

Supplement: S5 Fig — (A) Mouse OCT4 promoter sequence was analyzed by MethPrimer software. CpG methylation sites are individually shown in red. The OCT4 promoter region studied is encompassed by the green arrows (inner primers). (B) Nucleotide sequence of the OCT4 promoter region analyzed by bisulfite DNA sequencing. The 533 bp region starts approximately 500bp upstream of the transcription initiation site and contains 16 CpG sites. Different elements are highlighted in colors: green, specific primer sequences; red, CpG methylation sites; purple, open reading frame. (TIF) [file pone.0189131.s007.tif]

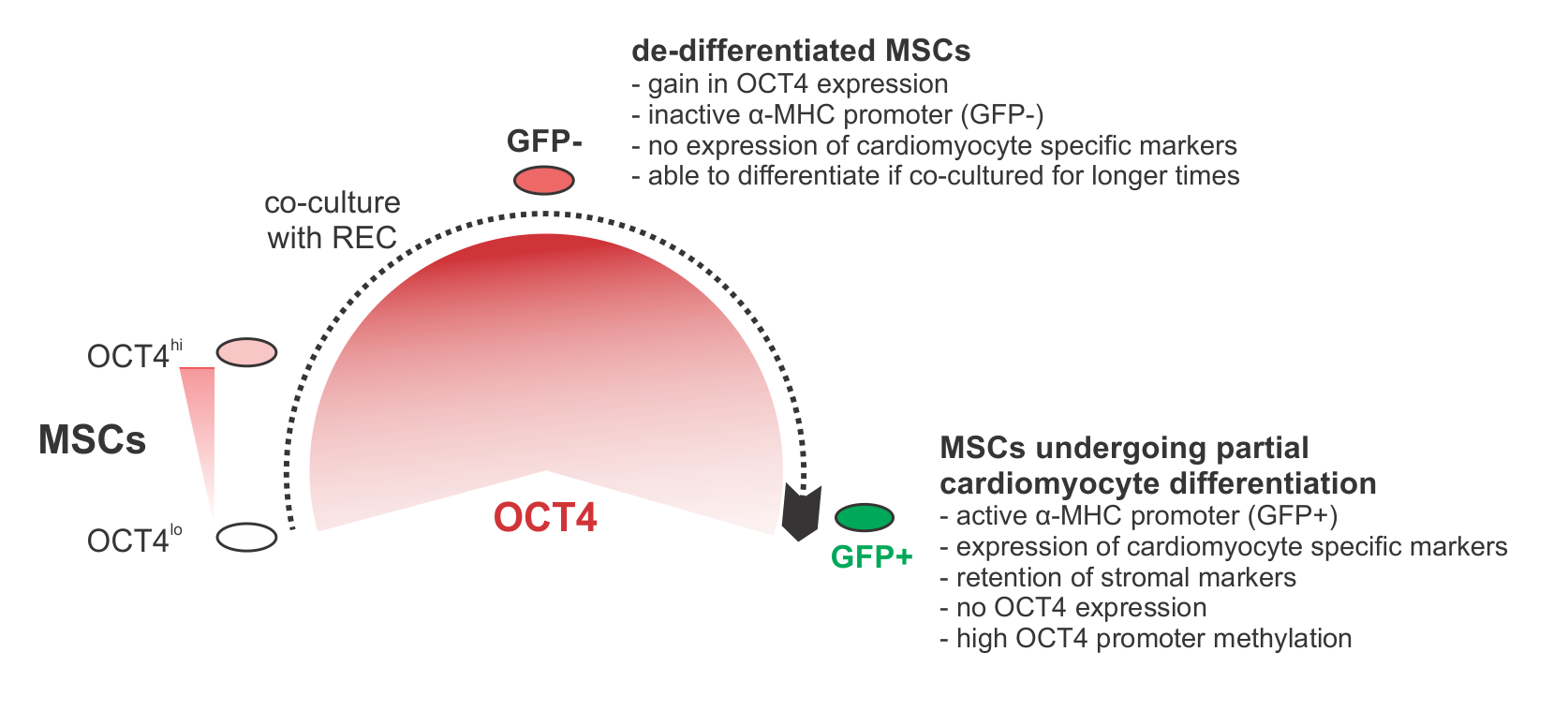

Supplement: S6 Fig — MSCs constitute a heterogeneous population of cells with a small range of OCT4 expression, which is related to their proliferation and multipotency capacity. Upon co-culture with REC, MSCs de-differentiate with a gain in OCT4 expression before being able to partially transdifferentiate into cardiomyocytes. MSCs starting with a high level of OCT4 expression completes this process within 5 days of co-culture, whereas de-differentiation takes longer for MSCs with low OCT4. Consequently, differences in the timing of reprogramming into cardiomyocytes may be due to cell heterogeneity among the MSCs. (TIF) [file pone.0189131.s008.tif]

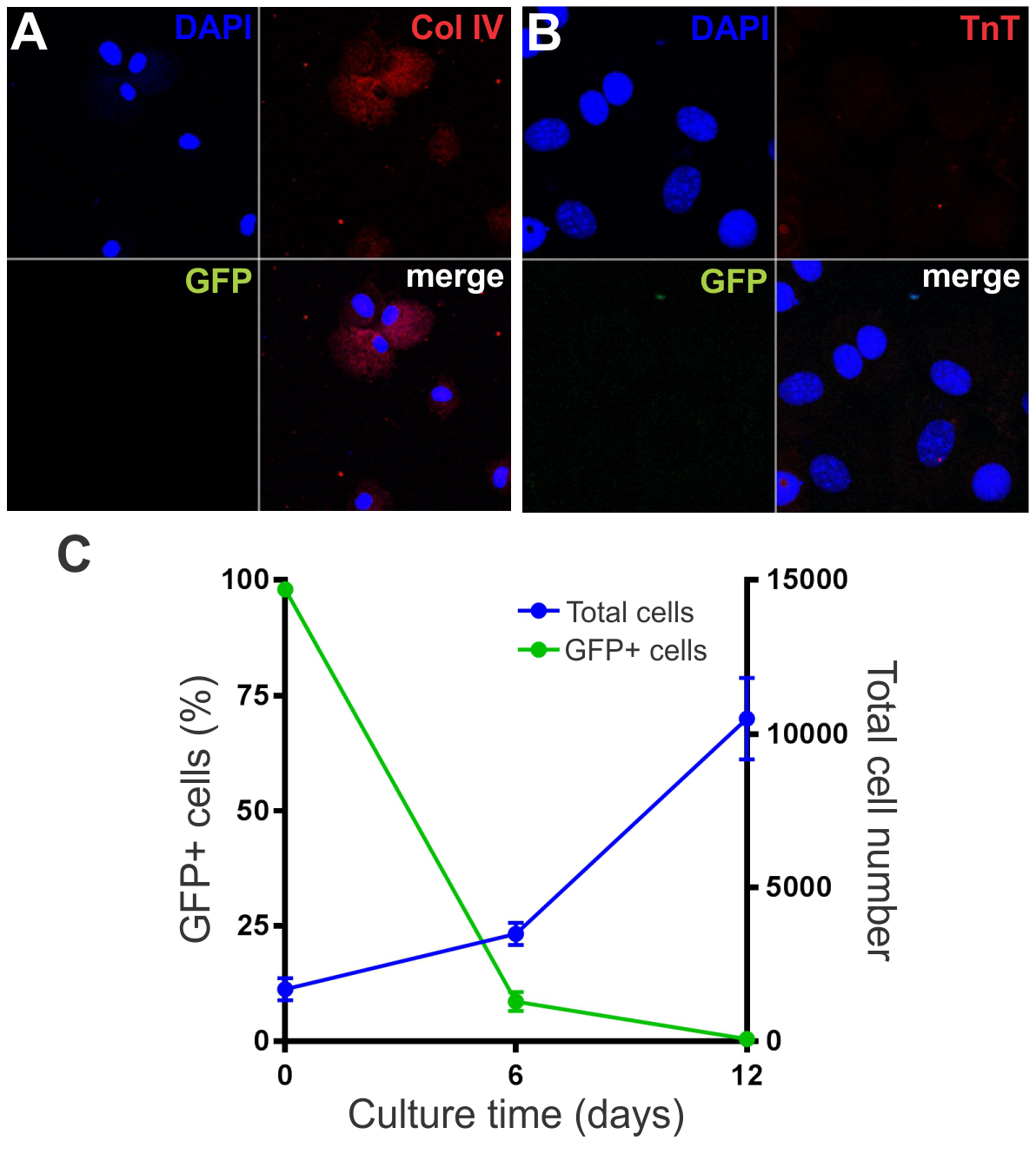

Supplement: S7 Fig — (A, B) GFP+ sorted cells express the stromal marker collagen type IV (Col IV) but lose the expression of the cardiac-specific protein troponin-T (TnT) after 12 days of culture in complete culture media. Images are representative of three independent experiments. (C) Growth curve and GFP expression on GFP+ sorted cells cultured under conventional conditions. Data represent mean±SD of three independent experiments. (TIF) [file pone.0189131.s009.tif]
